# Modified high-throughput Nile red fluorescence assay for the rapid screening of oleaginous yeasts using acetic acid as carbon source

**DOI:** 10.1186/s12866-020-01742-6

**Published:** 2020-03-14

**Authors:** Catarina Miranda, Sara Bettencourt, Tatiana Pozdniakova, Joana Pereira, Paula Sampaio, Ricardo Franco-Duarte, Célia Pais

**Affiliations:** 1 CBMA (Centre of Molecular and Environmental Biology), Department of Biology, University of Minho, Campus de Gualtar, 4710-057 Braga, Portugal; 2 Institute of Science and Innovation for Bio-Sustainability (IB-S), University of Minho, Braga, Portugal

**Keywords:** Yeasts, Intracellular lipids, Nile red, Volatile fatty acids, Single cell oils

## Abstract

**Background:**

Over the last years oleaginous yeasts have been studied for several energetic, oleochemical, medical and pharmaceutical purposes. However, only a small number of yeasts are known and have been deeply exploited. The search for new isolates with high oleaginous capacity becomes imperative, as well as the use of alternative and ecological carbon sources for yeast growth.

**Results:**

In the present study a high-throughput screening comprising 366 distinct yeast isolates was performed by applying an optimised protocol based on two approaches: (I) yeast cultivation on solid medium using acetic acid as carbon source, (II) neutral lipid estimation by fluorimetry using the lipophilic dye Nile red.

**Conclusions:**

Results showed that, with the proposed methodology, the oleaginous potential of yeasts with broad taxonomic diversity and variety of growth characteristics was discriminated. Furthermore, this work clearly demonstrated the association of the oleaginous yeast character to the strain level, contrarily to the species-level linkage, as usually stated.

## Background

The use of natural fats and oils for food consumption or as raw materials for biofuel production and bioenergy is creating a shortage of these materials, leading to problems associated with resource scarceness and environmental pollution. The production of single cell oils (SCOs), also called microbial lipids, could be one possible solution for these problems. Therefore, oleaginous microorganisms, the ones that are able to accumulate more than 20% of their dry cell weight as lipids in the form of droplets inside the cells [1, 2], have been the subject of increasing attention for their potential use in commercial production of oil for food, as well as for chemical and energy applications (reviewed in [3]). These SCOs include triacylglycerols (TAGs) that are the main central synthesized components inside microbial cells, but also glycolipids, phospholipids and steryl esters.

Oleaginous character is usually attributed to the microbial species-level, although some publications reported already some hints that it may vary at the strain-level. It is also known that oleaginicity can vary tremendously with the substrate and the growth conditions [4–8]. Yeasts stand out as the most favourable microorganisms to be used in oil production since they are simpler to handle in large-scale conditions [9], less susceptible to viral infections, and their use incorporates the ability to control bacterial contamination using lower pH values during growth [7]. Oleaginous yeasts have also several other important advantages to be considered: the ability to rapidly accumulate about 40 to 70% of lipids of their dry cellular biomass, the high growth rates and short life cycles, the presence of an enzymatic system suitable to the production and accumulation of lipids and also their capacity to tolerate and grow in a wide range of substrates [10]. Oil production using yeasts is affected by several factors, being the most relevant ones the carbon/nitrogen (C/N) ratio of substrate, the content and source of carbon, nitrogen and phosphorus, temperature, pH, growth stage, presence of alcohol and inhibitors (reviewed in [6]). When a nutrient such as nitrogen is depleted from the culture medium, a major shift in metabolism occurs, protein synthesis is depressed, and fatty acid synthesis is activated leading to its accumulation [4].

Volatile fatty acids (VFAs) are considered a promising carbon source for lipid production by oleaginous microorganisms to substitute other carbon sources, such as glucose, due to their shorter metabolic pathways and higher theoretical conversion efficiency [11]. In particular, acetic acid, a C2 compound with many applications, can be efficiently converted into lipids by oleaginous yeasts, being related to the acetyl-CoA metabolism. The creation of new fatty acids is entirely dependent on the continuous supply of acetyl-CoA, merely by the growth of oleaginous yeasts on acetic acid as carbon source [12]. After the uptake of this acid by the cells, it is converted into acetyl-CoA, being its excess used for intracellular lipid accumulation.

So far, several oleaginous yeasts have been studied, including Ascomyta (*Candida, Cyberlindnera, Geotrichum, Kodamaea, Lipomyces, Magnusiomyces, Metschnikowia, Trigonopsis, Wickerhamomyces, and Yarrowia*) and Basidiomycota (*Cryptococcus, Geomyces, Leucosporidiella, Pseudozyma, Rhodosporidium, Rhodotorula and Trichosporon*) [13–15], although the majority of genera and species are still to be analysed to verify oil content. One important aspect to consider is the fact that yeast taxonomy is continuously changing, so attention should be given to the species identification when considering one particular yeast species as oleaginous or not. Several conventional methods are available to quantify lipids produced by microorganisms, such as thin-layer chromatography (TLC), high-performance liquid chromatography (HPLC) and gas chromatography (GC) in combination with mass spectrometry (MS). Even though the reliability and credibility of these methodologies are high, lipid quantification usually implies high-costs and heavy equipment, and consequently screenings of large numbers of yeasts become complex and time-consuming, since lipid determination depends on several steps, including cell extraction, purification and concentration [16, 17]. Furthermore, there are three main limitations when using traditional techniques for quantifying lipids in several biological models: (1) the results are partly dependent on the cell wall disruption step; (2) the use of non-ecological and toxic organic solvents (e.g. chloroform and methanol); (3) no standardized extraction method is available to be applied to all oleaginous yeasts, since its efficiency varies greatly with the yeast species mainly due to their physiological properties. Hence, due to all these restrictions, an accurate, sensitive and rapid methodology for screening promising oleaginous strains with high capacity to accumulate lipids was needed. Several rapid screening methods have been proposed in the past, such as fluorimetric methods using Sudan black [18], Nile blue [19] and Nile red [20–23], colorimetric methods [24, 25], and, more recently the use of Fourier Transform Infrared (FTIR) spectroscopy [26, 27]. After this preliminary screening, more robust and detailed methods should be used to confirm and validate the obtained results.

Presently, the implementation of high-throughput tools for estimating the lipid content in oleaginous microorganisms have been exploited in microalgae and yeast species, yielding remarkable results [21–23, 28]. However, a great majority of yeast species have not yet been analyzed for lipid production and further strain selection and improvement are needed for commercial market applications. New high-throughput methods will allow to screen for a higher number of yeast isolates with suitable characteristics to utilize different substrates and different growth conditions, and to discover new oleaginous genera/species/strains that were not described so far in the literature.

In the present work we developed a protocol for the screening of oleaginous yeasts by using the Nile red fluorescence assay for measuring intracellular lipids, and introducing new key features: yeast cultivation in solid mineral medium and acetic acid as sole carbon source. A screening of around 370 yeast isolates for lipid production was accomplished in an easy, cheap and fast way, allowing the selection of new highly productive and promising yeast strains.

## Results

### Method validation

Eighteen yeast isolates belonging to different genera and species were randomly selected from our yeast bio-bank in order to evaluate the accuracy of published protocols and validate the methodology here proposed using acid acetic as carbon source for yeast growth on solid medium. For this, a correlation was established by comparing fluorimetric lipid detection with the traditional gravimetric determination, and the same fluorimetric quantification was used to compare lipid production by yeasts cultivated in broth and on solid media, as explained next.

### Lipid accumulation inside yeast cells determined by gravimetry and fluorimetry

First, neutral lipids produced by the eighteen yeast isolates were extracted gravimetrically using n-hexane as the organic solvent, and their percentage (w/w) was determined by weight difference. Table 1 summarizes the results obtained, expressed as “Lipid content (% (w/w))”, corresponding to an average value of three replicates and respective standard deviations. Data shows that about 72% of the yeasts (13 out of 18) were considered as oleaginous, and 28% (5 out of 18) were categorised as non-oleaginous, following the Ratledge [1] definition of oleaginous organisms as being the ones capable of accumulate more than 20% (w/w) of lipids, on dry cell basis. The highest values (≅ 60% (w/w)) were obtained for isolates V134 (*Apiotrichum brassicae*) and V194 (*Pichia kudriavzevii*). In this experiment two yeast species were included as being well known in the literature as either oleaginous (*Yarrowia lipolytica* - V011) or non-oleaginous (*Saccharomyces cerevisiae* – V673). Results here obtained are not in accordance with the literature, since the yeast *Y. lipolytica* V011 did not show high ability to accumulate intracellular lipids (11.9 ± 0.9% (w/w)), while *S. cerevisiae* isolate V673 obtained higher values (26.0 ± 3.6% (w/w)) than the ones achieved by *Y. lipolytica* and higher than the ones usually reported in literature [29], thus showing some oily capacity. However, the carbon source here used for yeast growth – acetic acid – is different than the one used commonly – glucose –, which could justify the differences found. This fact stresses that the oleaginous character of a strain should be attributed in close association with the carbon source in which the growth occurred.

**Table 1:** Relative fluorescence units (RFUs) determined by Nile red fluorimetric assay, and lipid content (% (w/w)) determined by gravimetric method after solvent extraction with n-hexane

Yeast ID	Yeast species	Lipid content (% (w/w))	RFUs (broth medium)	RFUs (solid medium)
V011	*Yarrowia lipolytica*	11.9 ± 0.9	9.9 ± 1.7	10.3 ± 1.7
V028	*Kazachstania humilis*	27.2 ± 1.7	30.3 ± 1.5	26.9 ± 2.5
V051	*Pichia kudriavzevii*	23.6 ± 5.8	14.8 ± 1.2	19.7 ± 1.3
V088	*Wickerhamomyces anomalus*	19.3 ± 2.1	17.6 ± 0.2	29.1 ± 2.7
V102	*Pichia fermentans*	28.4 ± 2.1	32.6 ± 0.1	31.9 ± 3.0
V124	*Cyberlindnera jadinii*	30.0 ± 2.4	20.4 ± 1.5	24.4 ± 0.5
V134	*Apiotrichum brassicae*	58.1 ± 2.3	71.1 ± 3.6	138.0 ± 18.3
V166	*Pichia fermentans*	22.4 ± 2.4	27.2 ± 0.5	41.4 ± 0.3
V188	*Pichia fermentans*	25.3 ± 0.6	25.9 ± 1.3	24.3 ± 1.3
V194	*Pichia kudriavzevii*	59.3 ± 0.6	85.1 ± 0.2	132.3 ± 7.1
V202	*Pichia kudriavzevii*	16.9 ± 4.5	10.5 ± 1.2	14.6 ± 0.5
V209	*Candida tropicalis*	21.7 ± 3.5	13.3 ± 2.0	54.9 ± 1.3
V211	*Geotrichum candidum*	13.3 ± 1.1	9.3 ± 1.4	6.7 ± 1.3
V213	*Metschnikowia pulcherrima*	29.6 ± 1.3	35.9 ± 1.1	42.2 ± 3.5
V247	*Geotrichum candidum*	16.1 ± 4.7	11.3 ± 1.2	5.6 ± 1.0
V535	*Saccharomycopsis vini*	26.6 ± 2.6	20.8 ± 2.2	25.2 ± 2.4
V673	*Saccharomyces cerevisiae*	26.0 ± 3.6	22.4 ± 1.9	18.7 ± 2.7
V674	*Saccharomycopsis fibuligera*	26.9 ± 0.6	19.0 ± 3.4	24.8 ± 3.2

Yeasts were cultivated during 120 h in broth and solid mineral media, with HAc 15 g/L as carbon source. Mean values of three replicates for RFUs (both in broth or solid media) did not differ significantly (*p* < 0.05)

In a second phase, and following Sitepu et al. [23] method, a Nile red fluorimetric assay was applied for yeast cultivated in broth and solid MAc media, being emitted fluorescence measured in the form of relative fluorescence units (RFUs) after 120 h of cell growth. Results are presented in Table 1, expressed as “RFUs (broth medium)” and “RFUs (solid medium)”. As expected, the capacity to accumulate lipids as determined by fluorimetry was not equal in all yeasts, and showed high variation among species, ranging from 9.3 ± 1.4 to 85.1 ± 0.2 RFUs when yeasts were grown in broth medium, and from 6.7 ± 1.3 to 138.0 ± 18.3 when solid medium was used. Considering these results, particular attention should be given again to isolates V134 (*A. brassicae*) and V194 (*P. kudriavzevii*), due to the highest RFUs obtained, in the same way as was observed using gravimetric method, especially when considering growth on solid medium for which the maximum RFUs values were 138 and 132, respectively. Other isolates also showed good capacity to accumulate lipids when grown in MAc - V102 (*P. fermentans*) and V213 (*Metschnikowia pulcherrima*) -, revealed by the obtainment of RFUs higher than 30 in both forms of cultivation. Again, *Y. lipolytica* V011 isolate had consistently low lipid production (RFUs: 9.9 ± 1.7 or 10.3 ± 1.7 considering broth and solid media, respectively) and in contrast, the yeast *S. cerevisiae* V673 showed some trend to accumulate lipids (RFUs: 22.4 ± 1.9 or 18.7 ± 2.7 considering broth and solid media, respectively) as detected by fluorimetric analysis. Once again, this difference seems to be related with the growth conditions that are known to deeply influence the lipid production more than the yeast species itself, as already shown before for *Y. lipolytica* [30].

### Correlation between gravimetric and Nile red fluorimetric methods

**Fig. 1:**
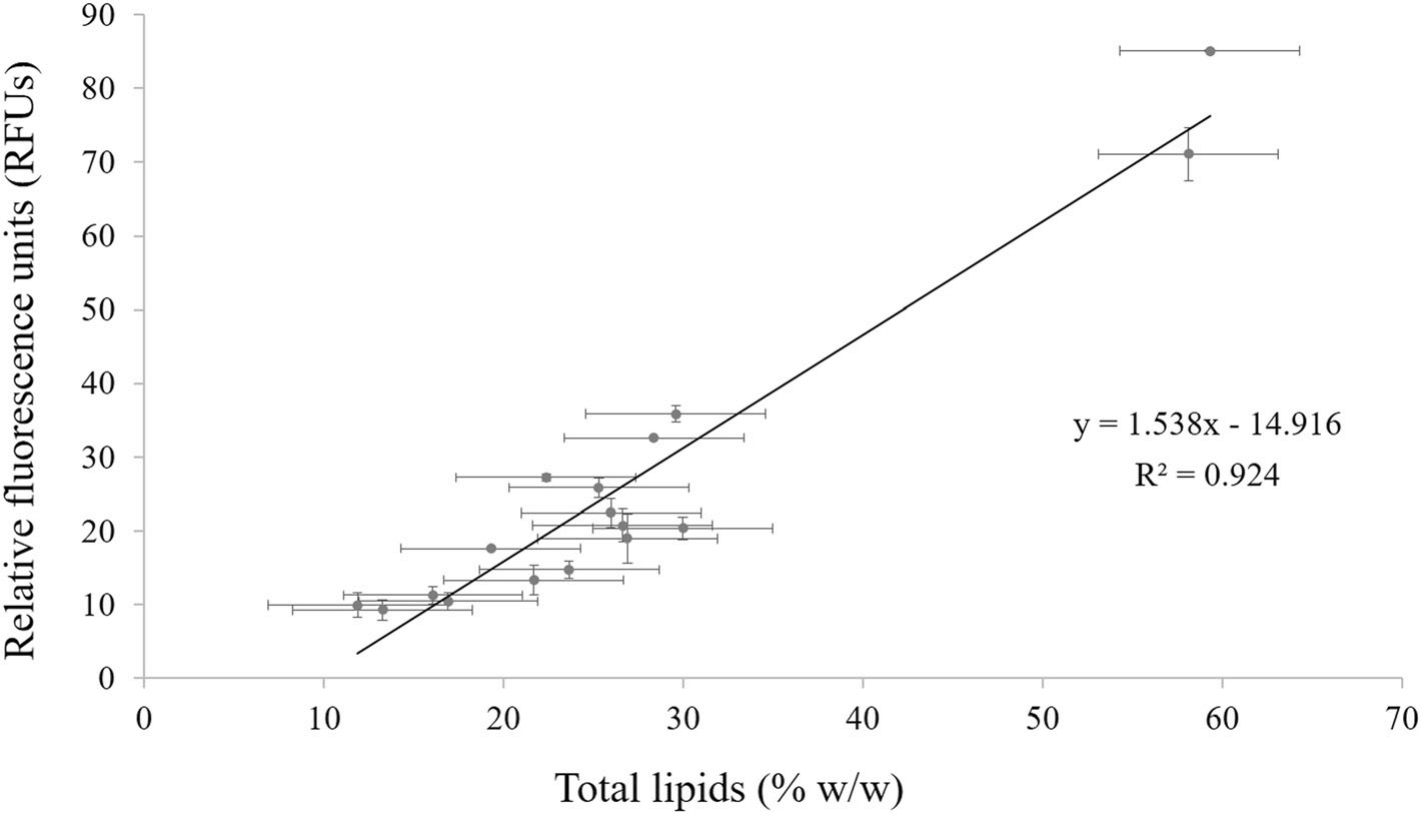
Correlation between fluorimetric and gravimetric method for 18 distinct yeasts cultivated in broth mineral medium with HAc 15 g/L, at pH 5.5 (R^2^ = 0.924). Data are mean ± standard deviation of three replicates

To validate the use of the proposed method, considering yeast growth on solid MAc medium and lipids quantified using Nile red fluorimetric approach, a correlation between experiments had to be performed, comparing lipid determination using both gravimetric and fluorimetric methods, after yeast growth in broth medium. For this, we assessed if the results obtained by the Nile red fluorimetric method were correlated with lipid quantification using the traditional gravimetric method after solvent extraction, considering the subgroup of 18 yeast isolates. Figure 1 presents the correlation obtained between methods, showing a very high correlation factor (R^2^ = 0.924), indicating the reliability of lipid estimation using Nile red fluorimetry for yeasts belonging to different species and genera, and with distinct ability to accumulate intracellular lipids. Using the equation present in Fig. 1 (y = 1.583 x – 14.916) it is possible to establish a correlation between the RFU values read in the fluorimeter and the % of lipids (w/w) determined by the gravimetric method. Considering the value proposed by Ratledge [1] – 20% of lipids accumulated – in order to consider a yeast as oleaginous, using the mentioned equation we determined that the corresponding value in terms of RFUs was around 16.

### Correlation between lipid production obtained after yeast growth on solid versus broth media

**Fig. 2:**
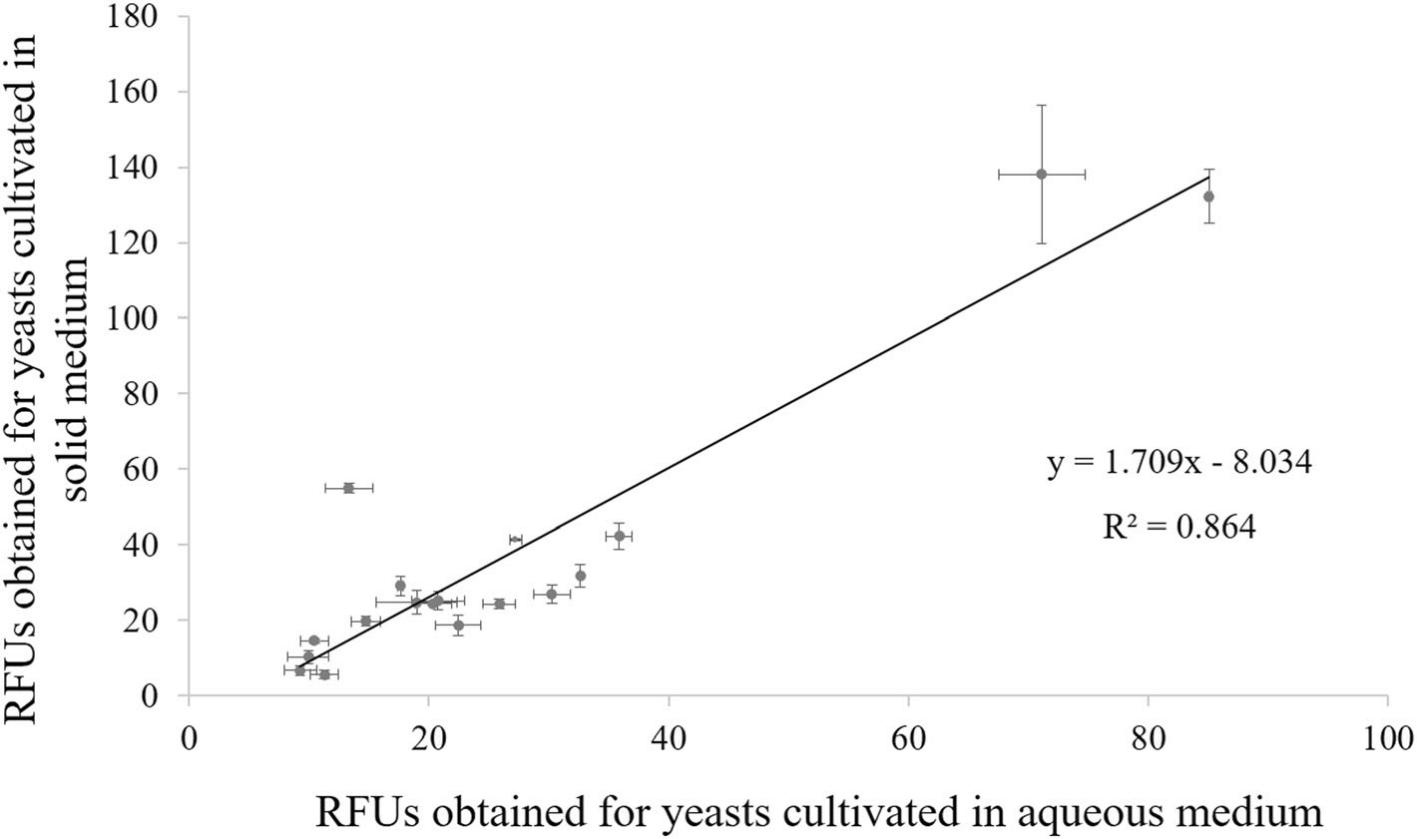
Correlation between relative fluorescence units (RFUs) for intracellular lipids obtained with yeasts cultivated in solid and broth media, composed of mineral medium with HAc 15 g/L as carbon source, at pH 5.5 (R^2^ = 0.864). Data are mean ± standard deviation of three replicates

After validation of the fluorimetric procedure for the estimation of lipid accumulation in yeasts (Fig. 1), the subsequent step was the evaluation of the possibility to cultivate yeasts on solid medium. Thus, yeasts were grown in two different conditions, using Petri dishes for solid medium and shake flasks for broth medium cultivation, using in both cases MAc medium. Lipid accumulation ability was estimated by fluorimetry (Table 1 and Fig. 2), and statistical analysis (one-way analysis of variance) displayed no statistical differences (*p* < 0.05; data not shown) between RFUs means of both experiments (broth and solid media) which points to the validity of using solid medium to perform yeast screening for lipid accumulation. In order to extent this analysis, a linear regression was established (Fig. 2), modulating the relationship between the two variables. Results showed the existence of a strong correlation (R^2^ = 0.864), meaning that cultivation of yeasts on solid medium doesn’t affect the determination of their lipid content.

### High-throughput screening of yeast isolates for lipid production: evaluation of yeast oleaginous character

After validation of the procedure, it was used for the screening of our 366 isolates. Using solid medium cultivation and Nile red fluorescence lipid determination we screened the yeasts for their capacity to produce SCOs. After 120 h, the lipid production was estimated by the Nile red fluorescence method in a 96-well dark microplate, and kinetic readings were performed during 20 min, being the maximum values of RFUs for each isolate determined after this time. This type of kinetic analysis accounts for individual variability since each yeast species presents its maximum fluorescence emission at a different time after Nile red incubation. With the single analysis performed at the same time for all the isolates, as was being performed before the protocol development by Sitepu et al. [23], maximum production capacity of strains evaluated during screenings would be overlooked. A total of 366 yeast strains was tested, from widely different environments, belonging to 54 genera and to 106 distinct species, both from Ascomycota and Basidiomycota phyla. In fact, for numerous yeast species, the information about their lipid production ability was still unknown.

**Fig. 3:**
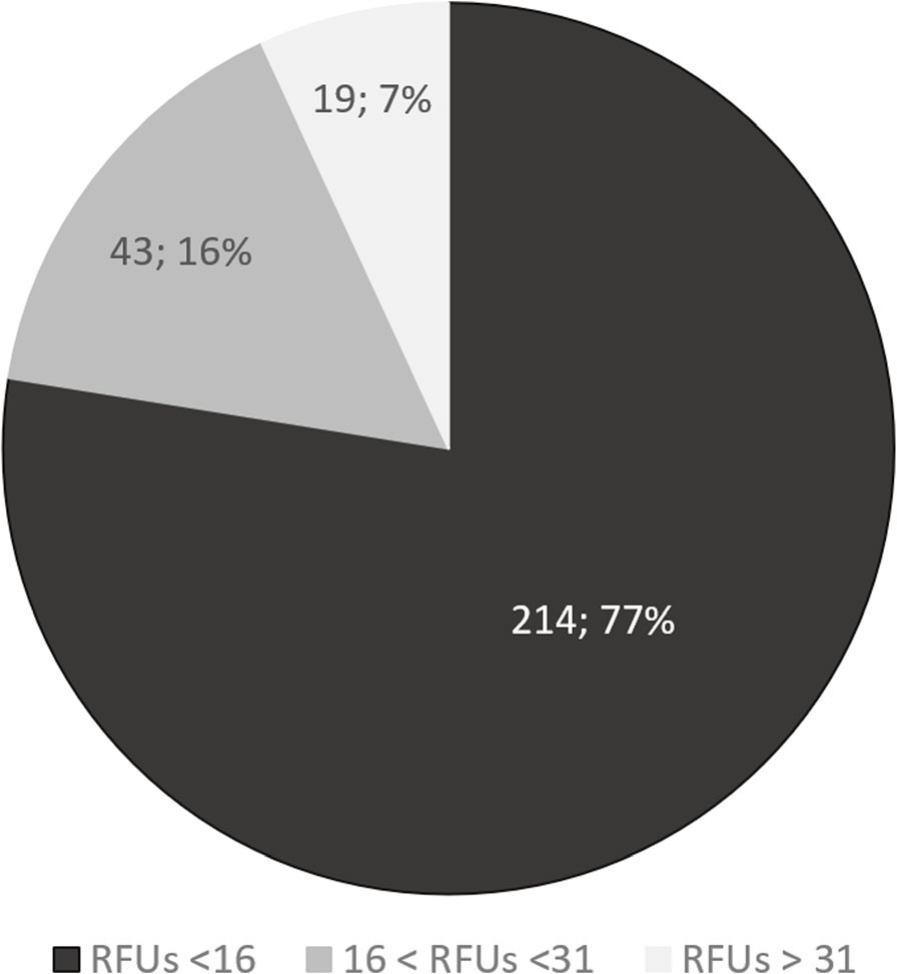
Yeast lipid synthesis ability, in terms of relative fluorescence units (RFUs), of 276 screened yeasts, as being able to grow in mineral medium with HAc 15 g/L, at pH 5.5. The first number inside the chart indicates the absolute number of isolates, and the second one the total percentage

Figure 3 summarizes the results obtained after the screening of all isolates using our proposed method. From the total 366 yeasts included in the screening, only 276 isolates were able to grow in HAc 15 g/L as carbon source, since for the other 90 isolates, no yeast colonies were detected on the Petri dishes, and therefore it was impossible to perform the fluorimetric assay. This fact is associated with the lack of capacity of these isolates to assimilate acetic acid as carbon source, especially in this concentration, as already referred by other authors [30–32]. Nevertheless, this carbon source seems to be the most appropriate to evaluate yeast oleaginicity potential [29, 31–34]. According to Fig. 3, it was possible to distribute isolates according to their lipid-accumulating ability. The highest number of isolates (214) were grouped as having RFUs below 16 (corresponding to less than 20% of lipids (w/w) when considering the equation of Fig. 1), indicative of their non-oleaginous character. This group, comprising 77% of the tested yeasts was not considered as good candidates to be used industrially and, therefore, it wasn’t exploited further in this work. Yeasts considered to have promising potential to produce lipids were grouped as having RFUs between 16 and 31 (43 isolates corresponding to 16% of the tested yeasts). These isolates show some capacity to produce lipids but not in quantities that lead to their use in industry. However, this should be the group of isolates to be tested in future works, for example varying the carbon sources of testing media and using different C/N ratios. The third and most important group in the context of this screening is the one containing isolates that obtained RFUs higher than 31 (corresponding to more than 30% of lipids (w/w) when considering the equation of Fig. 1), composed by yeasts considered to be highly oleaginous. This group (Table 2) is composed by 19 isolates, corresponding to 7% of the tested yeasts, achieving RFUs between 31 and 138. As one can conclude by the analysis of Table 2, the capacity of accumulating lipids was different and quite high for these oleaginous yeast species, with four isolates being capable to obtain RFUs values higher than 60. Isolates V134 and V194, belonging to species *A. brassicae* and *P. kudriavzevii*, respectively, were the two best promising oleaginous yeasts found in this screening, with quantified RFUs higher than 130. These two species are phylogenetically different, but behave identically regarding their oily capacity since their lipid contents were almost equal in the gravimetric analysis (≅ 60% (w/w), Table 1). Regarding yeast *A. brassicae*, also designated *Trichosporon brassicae*, its oleaginous capacity was already identified by Franklin et al. [35], who recorded a lipid content of 20.3% (w/w) for this yeast species. In comparison with the almost 60% (w/w) of lipids achieved in the optimisation experiment (Table 1), and 138 RFUs estimated for this yeast in the screening (Table 2), this may be the first study reporting the highest lipid content value for this species. In the case of yeast *P. kudriavzevii* V194, some studies have already stated high lipid contents for this ascomycete species as observed in this screening, which corroborates the oleaginicity often attributed to the genus *Pichia* [36, 37]. Within this genus, it was possible, for the first time to categorise two other yeast species as oleaginous, such as *P. fermentans* and *P. exigua* (Table 2), due to the inexistence of information in the literature about the oleaginous character of these two species.

**Table 2:** Relative fluorescence units (RFUs) of the 19 yeast species considered as being highly oleaginous (more than 30% (w/w) of their DCW in lipids) after the screening using the proposed innovative method^a^

Yeast ID	Yeast species	Phylum	RFUs
V134	*Apiotrichum brassicae*	Basidiomycota	138.0 ± 18.3
V194	*Pichia kudriavzevii*	Ascomycota	132.3 ± 7.1
V311	*Candida tropicalis*	Ascomycota	78.1 ± 2.5
V417	*Millerozyma farinosa*	Ascomycota	60.5 ± 1.9
V036	*Pichia exigua*	Ascomycota	56.3 ± 5.1
V343	*Candida tropicalis*	Ascomycota	55.5 ± 3.9
V209	*Candida tropicalis*	Ascomycota	54.9 ± 1.3
V447	*Hyphopichia burtonii*	Ascomycota	50.9 ± 4.4
V213	*Metschnikowia pulcherrima*	Ascomycota	42.2 ± 3.5
V114	*Candida tropicalis*	Ascomycota	41.9 ± 4.8
V166	*Pichia fermentans*	Ascomycota	41.4 ± 0.3
V473	*Candida palmioleophila*	Ascomycota	40.9 ± 1.6
V053	*Rhodotorula mucilaginosa*	Basidiomycota	39.0 ± 15.1
V139	*Candida tropicalis*	Ascomycota	38.9 ± 6.1
V418	*Trigonopsis cantarellii*	Ascomycota	38.1 ± 6.6
V342	*Geotrichum candidum*	Ascomycota	35.2 ± 2.0
V137	*Candida tropicalis*	Ascomycota	32.9 ± 2.1
V336	*Candida tropicalis*	Ascomycota	32.2 ± 3.6
V102	*Pichia fermentans*	Ascomycota	31.9 ± 3.0

^a^RFUs cut-off value for considering a yeast as oleaginous was 31, as obtained by the equation proposed in Fig. 1. RFUs values are mean ± standard deviation of three replicates

### Intraspecific variability of oleaginous character within yeasts: should oleaginous character be credited at strain level?

Besides distinct species, the present screening also included several yeast isolates of the same species in order to ascertain whether lipid accumulation may be strain dependent. Some examples of different yeast isolates belonging to the same species are described in Table 3 together with the respective RFUs obtained in the screening. For some species, considerable variations in lipid accumulation ability were observed, being possible to detect differences in the oleaginous potential at strain level, instead of species level as usually reported in the literature. For example, for *C. tropicalis*, the lipid accumulation capacity ranged from 2 to 78 RFUs, having the isolate V311 the highest (RFUs = 78) and the isolate V286 (RFUs = 2) the lowest lipid accumulation capacity. In the case of *G. candidum* species, it was possible to observe opposite capacities to accumulate lipids in the four isolates tested, as the yeast V342 (RFUs = 35) was considered oleaginous, the yeast V315 (RFUs = 28) was categorised as moderately oleaginous and the yeasts V395 and V249 (RFUs = 9; RFUs =1) were considered non-oleaginous. Considering *M. pulcherrima* and *P. fermentans*, the differences in oily capacity from distinct yeast strains were also observed, as the RFU values varied from 5 to 42 for the first species and 2 to 41 for the last one, respectively. No influence was found from the source of each strain isolation in the attribution of the oleaginous character (data not shown).

**Table 3:** Relative fluorescence units (RFUs) obtained by different isolates of the same yeast species^a^

Yeast ID	Yeast species	RFUs
V311	*Candida tropicalis*	78.1 ± 2.5
V209		54.9 ± 1.3
V338		20.9 ± 2.7
V286		2.4 ± 0.3
V342	*Geotrichum candidum*	35.2 ± 2.0
V315		27.7 ± 1.2
V395		9.1 ± 1.4
V249		1.4 ± 0.3
V213	*Metschnikowia pulcherrima*	42.2 ± 3.5
V556		6.8 ± 0.7
V566		5.4 ± 0.3
V166	*Pichia fermentans*	41.4 ± 0.3
V102		31.9 ± 3.0
V153		19.5 ± 1.7
V057		1.7 ± 0.2

^a^RFUs values are ± mean and standard deviation of three replicates

## Discussion

In the first part of this work, a protocol for the detection and estimation of lipids produced by yeasts was optimized. Using a group of eighteen distinct yeast isolates grown in a medium containing acetic acid as carbon source, it was possible to obtain a valid correlation between Nile red fluorimetric assay and the traditional gravimetric extraction method, which allowed to consider the fluorimetry as a suitable, accurate and reliable method to estimate the intracellular lipid production among several yeasts of distinct species and genera. The establishment of this correlation allowed the estimation of yeast lipid content by using only the fluorimetry approach, thus avoiding the tedious and time-consuming process associated with the gravimetric extraction. This correlation was also obtained before by other authors [21, 23, 38, 39], although either with a much smaller correlation factor than the one obtained in this work, or with a similar correlation factor but considering a lower number of isolates. Results show that strains with higher fluorescence also have higher lipid content, and vice versa, indicating that it is possible to predict the lipid content of each yeast by using only the fluorimetric approach also when using acetic acid as sole carbon source for yeast growth.

With the aim of validating the solid medium cultivation to grow yeasts in a high-throughput screening comprising several yeast isolates, a correlation was obtained between RFUs means of two different experiments: yeasts cultivated in shake flasks and cultivation in solid medium, using in both cases MAc medium. This is, to the extent of our knowledge, the first validation of this method using cultivation of yeasts on solid medium with the objective to screen for their SCOs accumulation properties, conjugating with a previously optimized procedure for the fluorescence quantification of lipid production. Some of the main advantages of this protocol are related to the fact that: (i) it is rapid, since it does not require the cell harvesting and washing step as in the fluorimetric analysis performed in broth medium; (ii) it requires less incubation space associated to the use of Petri dishes, in comparison with the shake flasks; (iii) it is less expensive, since the overall costs for solid medium cultivation are cheaper than for broth cultivation; (iv) it is less energy-demanding because it does not need a rotary shaker for incubation or a centrifugation step; (v) it is high-throughput, so that at least four to eight yeasts can be cultivated in a Petri dish, instead of single yeast cultivations in shake flask. This innovative procedure allows the high-throughput screening of a large number of isolates in a shorter amount of time and in a much easier way than using traditional methods.

After validation of the proposed method, it was used for the screening of the 366 yeast isolates. Our results discriminated 17 Ascomycetous species as high lipid producers, although the higher producer – *A. brassicae* – belongs to Basidiomycota phylum. Over the years, several yeast species were associated with oleaginous character, being this number higher than 40 already in 2012 [40] and continuing to grow during the last 10 years. In 2016, one report stated that already 70 species are identified as oleaginous [41]. These oleaginous yeasts are both from Ascomycota phylum (*Candida, Cyberlindnera, Geotrichum, Kodamaea, Lipomyces, Magnusiomyces, Metschnikowia, Trigonopsis, Wickerhamomyces,* and *Yarrowia*) and from Basidiomycota phylum (*Cryptococcus, Geomyces, Leucosporidiella, Pseudozyma, Rhodosporidium, Rhodotorula* and *Trichosporon*). Wang et al. [15] stated, using genome sequencing, that lipogenic capacity was acquired by fungi only after the divergence of Ascomycota, Basidiomycota, Chytridiomycota and Mucoromycota clades. In this way, no clear conclusion can be made about the comparison of lipid production between the two phyla. One aspect of notable importance is the fact that Basidiomycete yeast species are able to utilize a broader array of carbon sources than Ascomycete yeast species [42], and in this way the finding of good oleaginous candidates belonging to this phylum makes them perfect candidates to be used in industrial production, and in environmental projects using a wide array of substrates to produce lipids.

Even though a high oleaginous capacity was detected for the yeast *Hyphopichia burtonii* (V447) in the screening (RFU = 51), the cultivation of this yeast in solid MAc resulted in the formation of several hyphae invading the agar, thus increasing the complexity to collect cells and analyse their lipid accumulation. This species was already described as oleaginous in a previous report [35], although with no described information about its lipid content. Likewise, this screening also provided data about the oleaginous character of *M. farinosa, T. cantarelli* and *G. candidum* for the first time. Even though Ratledgle [43] has considered the yeast *G. candidum* as oleaginous, no data are available about its lipid content, making this screening the first study providing detailed information about oleaginicity capacity of this species. On the other hand, some of the yeast species reported as oleaginous in the literature were also found in this group: *A. brassicae, P. kudriavzevii, C. tropicalis, M. pulcherrima, C. palmioleophila* and *R. mucilaginosa* [8, 36, 44, 45].

Another surprising result was related to the fact that this screening did not categorise any *Y. lipolytica* isolate as oleaginous, even though this species is currently recognised as a model microorganism for lipid production [46]. Considering the five yeast strains tested, the obtained RFU varied from 4.6 to 11.4, indicating that none of the tested strains of *Y. lipolytica* was able to accumulate significant quantities of lipids, and therefore they were considered as non-oleaginous. Indeed, this allowed to demonstrate that the cited lipid content represents only one reported value for this particular species, and for instance when other strains of *Y. lipolytica* are cultivated under distinct conditions, they may present higher or lower lipid contents, as already shown [5, 7]. Moreover, during the growth of these five yeast strains, the production of several hyphae in the solid MAc was observed. This is actually a typical behaviour of this dimorphic yeast cultivated under nitrogen-limited conditions, and is often associated with a defence mechanism to some adverse conditions [47]. Actually, although *Y. lipolytica* has been referred as oleaginous, there are no reports of high lipid accumulation. This species has been widely used as a classic species because its genome has been sequenced early, which allowed its engineering towards an higher lipid accumulation [3, 30, 41, 48]. Nowadays, many yeast genomes are available, so other yeast species can be used in this kind of studies.

Results clearly show that lipid accumulation varies among strains of the same species, therefore yeast oleaginous potential should be attributed at the strain level, instead of species-level linkage as usually stated in the literature. This idea was already reported in other studies [4, 5, 7, 48]. For example, in the study carried out by Garay et al. [41], two different strains of *R. mucilaginosa* were tested and their lipid contents varied from 20 to 45% of lipids. Sitepu et al. [23] included four independent isolates of *Rhodosporidium diobovatum*, which presented lipid contents varying from 21 to 41% (w/w). Likewise, Katre et al. [5] reported lipid contents in a range from 22 to 31% (w/w) for five isolates of *Y. lipolytica* tested. Even though several examples have been reported during the last years, it is still standard practice to describe yeast species as oleaginous or non-oleaginous, directing to the (erroneous) fact that all strains of that species should have the same behaviour.

## Conclusions

The easy, cheap and fast protocol here optimised was based on using solid medium cultivation and subsequent neutral lipid estimation by fluorimetric method, instead of broth medium and traditional gravimetric method determination. This high-throughput protocol proved to be adequate for the screening of large number of yeasts as a preliminary step for the selection of new candidates to be used at industrial level.

Our work discriminated the oleaginous potential of yeasts with broad taxonomic diversity and variety of growth characteristics. We highlighted strains of the species *Apiotrichum brassicae* and *Pichia kudriavzevii* as good candidates to accumulate lipids using acetic acid as carbon source.

## Methods

### Yeast isolates and culture conditions

Three hundred and sixty-six yeast natural isolates were used in this study. They were isolated from different environmental sources, such as fruit and vegetable wastes, fermented beverages, soils, etc. (Supplementary Data S1), in the ambit of several research projects previously developed, identified, and stored in our research center. From this group, 18 isolates were randomly chosen and used to validate a new screening protocol. To confirm the species identification of the yeasts (the ones chosen randomly to validate the protocol – Table 1 –, and the ones catalogued as oleaginous after the screening – Table 2), the internal transcribed spacer (ITS) region was sequenced and DNA sequences were deposited in NCBI GenBank database under the Accession Numbers depicted in Supplementary Data S2. To each isolate, a code composed of “V” plus three digits was attributed, as detailed in Table 1. All yeasts were stored at − 80 °C in cryo-tubes with 1 mL glycerol (30% v/v).

Prior to all experiments, yeasts were revived from cryo-preserved stocks, collecting a portion of cell biomass to Yeast Extract Peptone Dextrose (YPD) medium (1% yeast extract (w/v), 2% peptone (w/v), 2% dextrose (w/ v)), supplemented with agar 2% (w/v), and incubated at 30 °C.

For quantification of yeast lipid accumulation, a mineral medium with acetic acid was used (MAc: acetic acid 15 g/L, yeast extract 0.75 g/L, MgSO_4_.7H_2_O 1.5 g/L, KH_2_PO_4_ 0.4 g/L, (NH_4_)_2_SO_4_ 0.1 g/L, pH 5.5), previously shown to be adequate to the production of SCOs by yeasts [31]. Acetic acid was microfiltered to avoid heat-induced volatilization and added to autoclaved medium. When experiments were to be performed on solid medium, 2% (w/v) agar was added to the MAc medium.

Yeasts were pre-cultured in YPD medium and incubated during 24 h (170 rpm, 30 °C). After this period, cultures were harvested by centrifugation, washed once in MAc medium, and optical density (OD) was measured at 640 nm and adjusted to 0.6. For lipid accumulation analysis regarding yeasts cultivated in broth medium, yeasts were incubated in 40 mL MAc medium in 100 mL Erlenmeyer flasks on a rotary shaker at 170 rpm, 30 °C, pH 5.5, for 120 h. For solid medium cultivations, yeast cells were pipetted to a Petri dish with MAc medium, and plates were incubated at 30 °C, pH 5.5, for 120 h. For both cultivations, three replicates were performed to ensure the reproducibility and reliability of the results.

### Lipid quantification

#### Experimental setup

To estimate the lipid accumulation ability of yeasts, the protocol described by Sitepu et al [23] was used with the following modifications: i) acetic acid as carbon source; ii) yeast cultivation on solid medium. In order to validate the use of this method to determine lipid quantities produced by yeasts, the existence of a correlation between fluorimetric and gravimetric methods, as well as the comparison of the cultivation of yeasts in solid or broth media was tested, using MAc medium. After 120 h of cultivation in broth medium, yeast biomass was determined and lipids were quantified by gravimetry and fluorimetry. For yeasts growing on solid medium, lipid synthesis ability was determined only by fluorimetry.

### Dry cell weight and gravimetric determination of lipid content

Dry cell weight was determined by transferring 40 mL of yeast cultures to 50 mL conical tubes, centrifuging (5000 rpm, 2 min) and washing with sterile deionised water, until removal of all the growth medium. Cell pellets were transferred to a previously weighed 2 mL microtube, frozen in liquid nitrogen and stored overnight at − 80 °C. Following, cells were freeze-dried (Alpha 2–4 LD plus, BIOBLOCK scientific, CHRIST®) for 48 h, 0.010 mbar at − 83 °C, and lyophilised cells were maintained in the desiccator until gravimetric analysis. Yeast dry cell weight was determined by weight difference after lyophilisation and expressed in terms of g biomass/L culture.

For the gravimetric determination of lipid content approximately 20 mg of lyophilised cells were weighed to 2 mL screw cap tubes with 800 mg of glass beads (diameter 0.25–0.50 mm, RETSCH®). Cells were homogenised in different series using a FastPrep®FP120 Cell disrupter for 30 s, 8 times, speed 6.5 m/s with 60 s interval on ice. A total of 3 mL of n-hexane (99.0% of purity, PANREAC®) were used to extract lipids and 0.6 mL 0.9% NaCl was added to 15 mL glass tubes to solubilize cellular compounds, being the solution then mixed in a vortex for 40 s and centrifuged at 3018 rpm (1020 g) for 3 min. After centrifugation, the superior phase was filtered to a 4 mL pre-weighed amber vial and evaporated under a low stream of nitrogen. Vials were dried at 50 °C for 1 h, and weighted. The lipid content was determined by dividing the difference of the vial weight by the mass of lyophilised cells previously weighed for each screw cap tube, and expressed in terms of gram of lipid per gram of DCW (% (w/w)). The efficacy of each extraction was evaluated and corrected by using commercial oil as a standard control.

### Lipid content estimation using Nile red fluorimetric assay

Regarding the fluorimetric assay, the procedure for yeast grown in broth and on solid media was similar, being cells washed and final OD_640_ adjusted to 1.0 using PBS 1x, since it represents the adequate cell density to obtain optimum fluorescence measurement [23]. Then, a volume of 817 μL of cells were mixed with 83 μL DMSO:PBS 1x (1:1 (v/v)) in 1.5 mL microtubes, and 5 μg of Nile red (Sigma-Aldrich®) diluted in acetone were added to the mixture. In a dark environment, 200 μL of the suspension were transferred, in triplicate, to 96-well black microplates (Corning®, Cat.#3915), being the fluorescence intensity instantly read in a microplate fluorimeter (Fluoroskan Ascent FL, Thermo Scientific®), with the following parameters, adapted from [23]: initial fluorescence excitation at 530 nm and emission at 590 nm, kinetic readings for 20 min with 60 s interval and 30 s of shaking. Independent control samples were prepared consisting of Nile red diluted in acetone, PBS 1x and DMSO:PBS 1x (1:1 (v/v)). Blank samples were prepared by substituting the cell suspension by the same volume of PBS 1x. Cells auto fluorescence was also measured substituting Nile red solution by pure acetone. In addition, biological control samples (yeast suspensions whose lipid concentration was previously determined) were also prepared and included in each microplate to evaluate consistency between readings. After kinetic readings, maximal emission values were determined and fluorescence data were corrected for variation in cell density by dividing the RFUs by background OD_640_ values and then by subtracting the mean of blank values.

### High-throughput screening of lipid producing yeasts

After a strong correlation was obtained between Nile red fluorescence method using solid and broth media, the former one was used in a high-throughput screening of a total of 366 yeast strains, in order to evaluate SCOs production, using solid MAc media, and the conditions described previously (“Experimental setup”).

### Data analysis

All values are means of three independent biological experiments. Statistical analyses were performed using Data Analysis Pack in Excel®. RFUs means were compared between broth and solid media, and also with the mean percentage of lipids determined by gravimetric analysis and evaluated using one-way analysis of variance (ANOVA).

## Supplementary information

**Additional file 1.**

**Additional file 2.**

## Data Availability

The datasets used and/or analysed during the current study are available from the corresponding author on reasonable request.

## Abbreviations

C/N: Carbon/nitrogen
DCW: Dry Cell Weight
FTIR: Fourier Transform Infrared
GC: Gas chromatography
HAc: Acetic acid
HPLC: High-performance liquid chromatography
MS: Mass spectrometry
RFUs: Relative Fluorescence Units
SCOs: Single cell oils
TAGs: Triacylglycerols
TLC: Thin layer chromatography
VFAs: Volatile fatty acids
